# Repeatedly Evolved Host-Specific Ectosymbioses between Sulfur-Oxidizing Bacteria and Amphipods Living in a Cave Ecosystem

**DOI:** 10.1371/journal.pone.0050254

**Published:** 2012-11-29

**Authors:** Jan Bauermeister, Alban Ramette, Sharmishtha Dattagupta

**Affiliations:** 1 Geomicrobiology and Symbiosis Group, Courant Research Center Geobiology, University of Göttingen, Göttingen, Germany; 2 HGF-MPG Group for Deep-Sea Ecology and Technology, Max Planck Institute for Marine Microbiology, Bremen, Germany; University of Vienna, Austria

## Abstract

Ectosymbioses between invertebrates and sulfur-oxidizing bacteria are widespread in sulfidic marine environments and have evolved independently in several invertebrate phyla. The first example from a freshwater habitat, involving *Niphargus ictus* amphipods and filamentous *Thiothrix* ectosymbionts, was recently reported from the sulfide-rich Frasassi caves in Italy. Subsequently, two new *Niphargus* species, *N. frasassianus* and *N. montanarius*, were discovered within Frasassi and found to co-occur with *N. ictus*. Using a variety of microscopic and molecular techniques, we found that all three Frasassi-dwelling *Niphargus* species harbor *Thiothrix* ectosymbionts, which belong to three distinct phylogenetic clades (named T1, T2, and T3). T1 and T3 *Thiothrix* dominate the *N. frasassianus* ectosymbiont community, whereas T2 and T3 are prevalent on *N. ictus* and *N. montanarius*. Relative distribution patterns of the three ectosymbionts are host species-specific and consistent over different sampling locations and collection years. Free-living counterparts of T1–T3 are rare or absent in Frasassi cave microbial mats, suggesting that ectosymbiont transmission among *Niphargus* occurs primarily through inter- or intraspecific inoculations. Phylogenetic analyses indicate that the *Niphargus-Thiothrix* association has evolved independently at least two times. While ectosymbioses with T1 and T2 may have been established within Frasassi, T3 ectosymbionts seem to have been introduced to the cave system by *Niphargus*.

## Introduction

Symbioses are vital for virtually all living organisms. They were critical for the origin and diversification of Eukaryotes and remain a major driving force in evolution, as they induce diverse physiological, morphological, and developmental modifications in the species involved [1]. Symbioses between invertebrates and chemosynthetic (e.g. sulfur- or methane-oxidizing) bacteria are of particular ecological importance in the marine environment, where they have evolved independently in at least seven metazoan phyla [2]. Many invertebrates living in sulfide-rich marine habitats, such as close to deep-sea hydrothermal vents, cold seeps, and in organic-rich coastal sediments, harbor sulfur-oxidizing bacteria on their body surfaces [2]–[3]. Although the animals are exposed to diverse free-living microbial communities and therefore susceptible to colonization by many opportunistic, non-specific surface-dwellers [4], many of them have established long-term and specific relationships with only few selected sulfur-oxidizing bacteria [5]–[10]. Most of these ectosymbionts belong to distinct groups within the epsilon- and gammaproteobacterial subdivisions. In particular, bacteria within the families Thiovulgaceae and Thiotrichaceae seem to have evolved an enhanced ability to establish ectosymbioses [3].

*Thiothrix* bacteria (family Thiotrichaceae) have been found as ectosymbionts on the marine amphipod *Urothoe poseidonis* [11] and on the freshwater amphipod *Niphargus ictus* [12]. The latter lives in sulfide-rich waters of the Frasassi caves (central Italy), which have been formed by sulfuric acid-driven limestone dissolution and contain an underground ecosystem sustained by chemoautotrophy [13]. Thick mats of filamentous sulfur-oxidizing epsilon- and gammaproteobacteria cover many of the cave water bodies [14]–[15]. *Thiothrix* are abundant in these microbial mats, but the ectosymbionts of *N. ictus* are distinct from most of the *Thiothrix* bacteria found in the free-living communities [12].

At the time this symbiosis was discovered, *N. ictus* was reported to be the only amphipod species inhabiting the Frasassi cave ecosystem [13], [16]. However, subsequent molecular and morphological investigations revealed the presence of two additional species [17], which were named *Niphargus frasassianus* and *Niphargus montanarius* [18]. Phylogenetic analyses suggested that the three *Niphargus* species most likely invaded the cave system independently [17]. *N. frasassianus* and *N. montanarius* have so far never been observed to co-occur, but each of them has been found in sympatry with *N. ictus* at some locations within the Frasassi caves.

Host-related factors are considered to play a decisive role in ectosymbiont selection and maintenance [19]–[20]. It has recently been shown that stilbonematid nematodes of two different genera living together in the same coastal marine sediments harbor distinct bacterial ectosymbiont phylotopes [10]. The *Niphargus* assemblage in Frasassi provided an opportunity to examine ectosymbiont specificity within partially sympatric, heterospecific members of the same invertebrate genus. In this study, all three Frasassi-dwelling *Niphargus* species were examined for *Thiothrix* ectosymbionts using a combination of Scanning Electron Microscopy (SEM), 16S rDNA sequencing, Fluorescence *In Situ* Hybridization (FISH), Automated Ribosomal Intergenic Spacer Analysis (ARISA), and nested-PCR. FISH was further used to inspect Frasassi microbial mats for free-living counterparts of the symbionts, and nested-PCR assays served for detecting symbiont dispersal cells. We report on three distinct *Thiothrix* ectosymbionts that are partially shared but yet distributed in a host species-specific manner among the *Niphargus*.

## Materials and Methods

### Sample collection & *Niphargus* species identification

**Figure 1:**
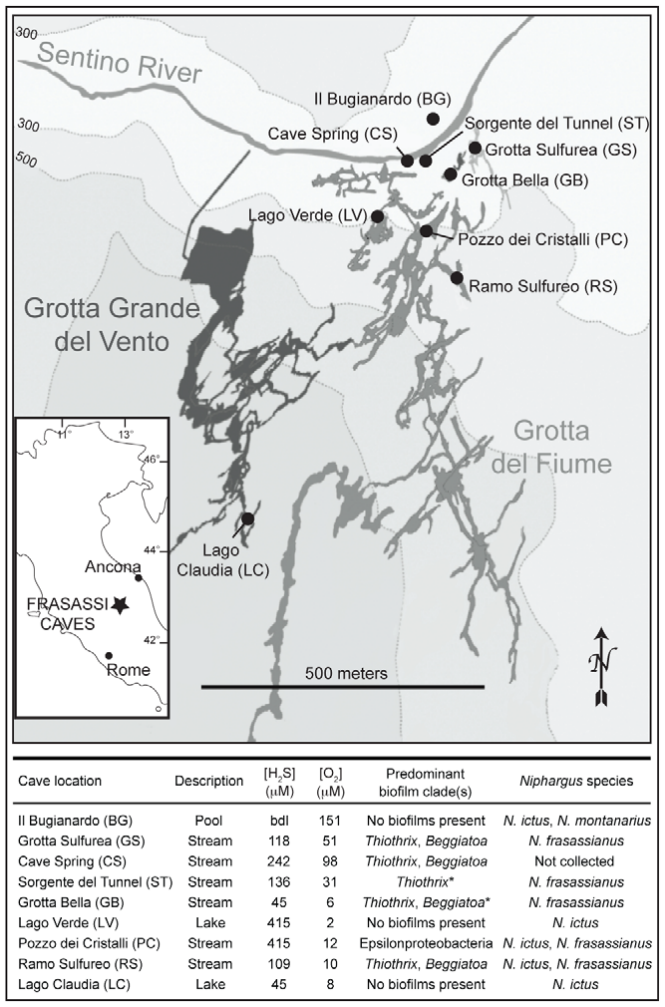
Map of the Grotta Grande del Vento-Grotta del Fiume complex of the Frasassi caves. Black circles in main map mark sample collection sites. Geochemistry data have been reported earlier by [17]. Predominant biofilm clade determinations are based on FISH results [15], except for those marked with *, which were identified based on morphology [14]. bdl = below detection limit. Base map courtesy of the Gruppo Speleologico CAI di Fabriano.

*Niphargus* specimens were collected in January and May–June 2008, May–June 2009, July and October 2010, and March 2011 from within the Frasassi Grotta Grande del Vento-Grotta del Fiume complex at eight different cave locations (Il Bugianardo (BG), Grotta Sulfurea (GS), Sorgente del Tunnel (ST), Grotta Bella (GB), Lago Verde (LV), Pozzo dei Cristalli (PC), Ramo Sulfureo (RS), and Lago Claudia (LC); Figure 1). All sites were accessed via technical spelunking routes.

*Niphargus* species were determined in the field based on morphological characters described in [17] and [18]. Individuals were caught using small fishing nets and forceps as appropriate. Specimens for SEM were collected into falcon tubes filled with cave water. They were later transferred to individual eppendorf tubes filled with a 2.5% glutaraldehyde solution made either in phosphate buffered saline (PBS) or in filter-sterilized cave water, and stored at 4°C until analysis. Samples for clone library construction, FISH, ARISA, and nested-PCR assays were collected into individual eppendorf tubes filled with RNAlater® (Ambion/Applied Biosystems, Foster City, CA, USA) and stored at −20°C until further analysis.

Microbial mat samples were obtained from Frasassi cave locations GS, ST, GB, PC, and RS in May–June 2009, and from locations Cave Spring (CS), GB, and RS in October 2011. They were collected into falcon tubes using sterile pipettes, preserved in 4∶1 parts of RNAlater® within 4 h of collection, and stored at −20°C until analysis.

### Scanning Electron Microscopy (SEM)

Two *N. frasassianus* individuals (locations GB and PC, June 2009), nine *N. ictus* individuals (location BG, June 2009 (1×), October 2010 (5×); location LC, May 2009 (1×); location LV, July 2010 (2×)), and one *N. montanarius* individual (location BG, June 2009) were investigated for *Thiothrix* epibionts using SEM. Whole specimens were sequentially dehydrated in ethanol concentrations from 30% to 90%, with a final dehydration in hexamethyldisilazane (SIGMA-ALDRICH, Munich, Germany) for 5–10 min. They were mounted on carbon-coated aluminum sample holders, sputtered with gold-palladium (11 nm thickness), and examined with a LEO 1530 GEMINI field emission SEM (Zeiss, Göttingen, Germany).

### DNA extraction

*Niphargus* appendages (legs and antennae) were dissected under a stereomicroscope. DNA extracts of *Niphargus* specimens collected in 2008 had previously been obtained from only two legs per individual (one gnathopod and one pereopod; cf. [17]). In order to increase the chance of gathering DNA from *Thiothrix* bacteria associated with *Niphargus*, DNA extractions for specimens collected from 2009 to 2011 were conducted with all appendages on one side of the *Niphargus* body. All extractions were performed using the DNeasy Blood & Tissue Kit (QIAGEN, Hilden, Germany), following the manufacturer's instructions (starting with an overnight treatment with Proteinase K, followed by DNA precipitation and purification). Microbial mat DNA was extracted using the PowerSoil DNA Isolation Kit (MO BIO Laboratories, Carlsbad, CA, USA) according to the manufacturer's instructions.

### 16S rDNA sequencing

16S rDNA clone libraries were obtained from five *N. frasassianus* samples (location GB, June 2008; location PC, May 2008; location RS, June 2008, May 2009; location ST, May 2009), two *N. ictus* samples (location BG, January 2008; location LC, May 2009), two *N. montanarius* samples (location BG, January 2008, June 2008), and one Frasassi microbial mat sample (location ST, May 2009). DNA was PCR-amplified using the bacterial domain-specific forward primer 27F and the universal reverse primer 1492R (both [21]; see Table S1 for sequences of all primers used in this study). The PCR mixture (50 µL) contained 1× ammonium buffer (Bioline, Luckenwalde, Germany), 5 mM MgCl_2_ (Bioline), 0.2 mM dNTP mix (SIGMA-ALDRICH), 15–30 ng of extracted DNA (quantified by a ND-1000 Nanodrop, PEQLAB Biotechnology, Erlangen, Germany), 1.25 units of BioTaq DNA polymerase (Bioline), and 500 nM of each primer. PCR was performed in a SensoQuest LabCycler (SensoQuest, Göttingen, Germany), with an initial denaturation at 94°C for 3 min, followed by 30 cycles of 94°C for 1 min, 50°C for 25 s, 72°C for 2 min, and a final extension at 72°C for 5 min. PCR products were checked on a 1% agarose gel. Bands of the correct size were excised and extracted using the QIAquick Gel Extraction Kit (QIAGEN). 16S rDNA fragments were cloned into pCR®4-TOPO® plasmids used to transform chemically competent One-Shot® MACH1™ *E. coli* cells (TOPO TA Cloning® Kit, Invitrogen, Darmstadt, Germany) according to the manufacturer's instructions. Colonies containing inserts were isolated by streak-plating onto LB agar mixed with 50 µg/mL ampicillin. Plasmid inserts were screened using colony PCR with M13F forward and M13R reverse primers. Colony PCR products of the correct size were purified using the QIAquick PCR purification kit (QIAGEN) and sequenced at the Göttingen Center of Molecular Biology using the plasmid-specific primers T3 and T7. Sequences were assembled using CodonCode Aligner version 3.7.1.1 (CodonCode Corporation, Dedham, MA, USA) and manually checked for ambiguities. They were screened for chimeras using Bellerophon version 3 [22]. Putative chimeras were excluded from subsequent analyses. A total of 144 non-chimeric 16S rDNA sequences were submitted to GenBank (accession numbers JN983537–JN983680).

### Phylogenetic analysis of 16S rDNA clone library sequences

**Figure 2:**
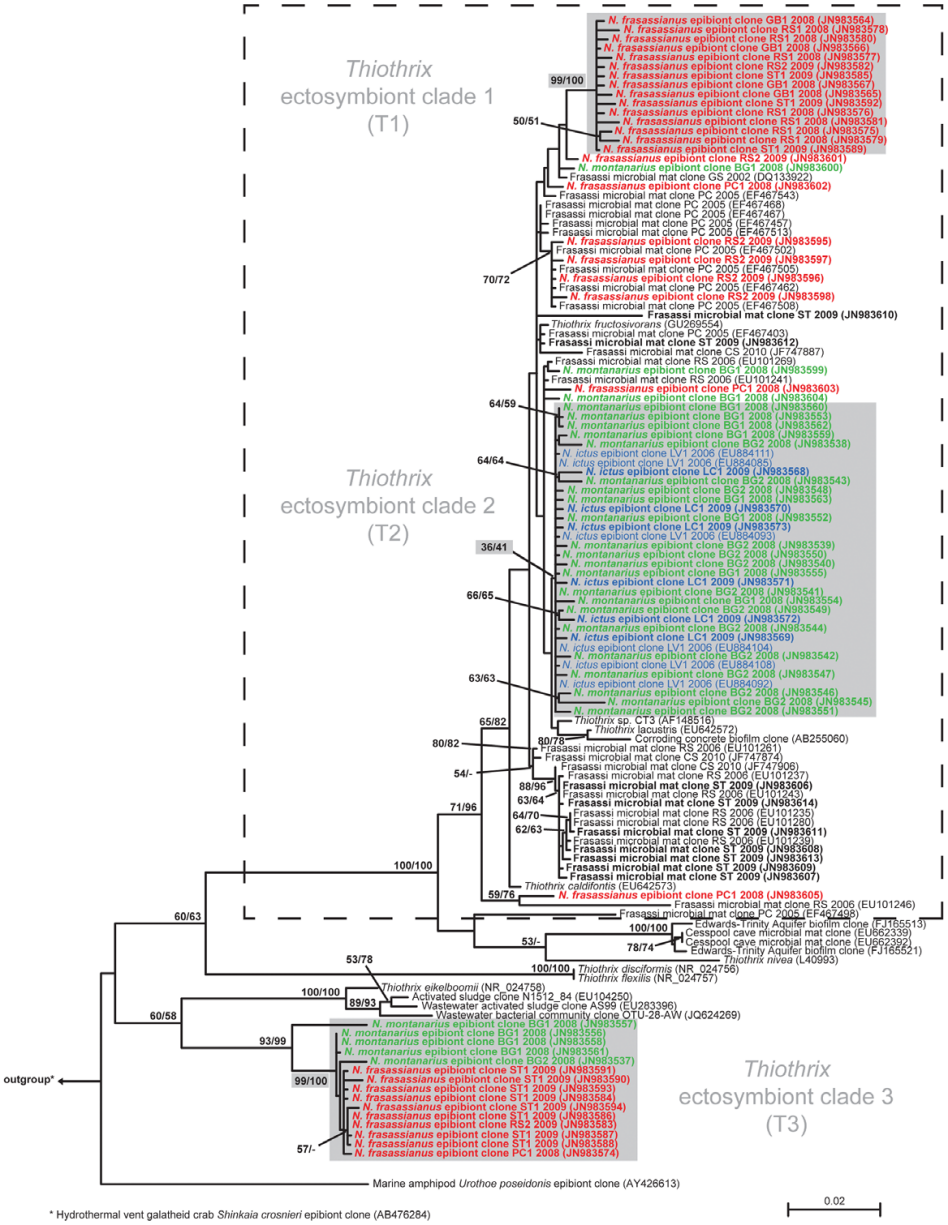
16S rDNA Maximum Likelihood phylogenetic tree of cultivated and uncultivated *Thiothrix* species. Sequences obtained in this study are in bold face, and clone names indicate the sampling location and year (BG = Il Bugianardo, ST = Sorgente del Tunnel, GB = Grotta Bella, LV = Lago Verde, PC = Pozzo dei Cristalli, RS = Ramo Sulfureo, LC = Lago Claudia). Different numbers after cave name abbreviations indicate *Niphargus* individuals collected from the same cave location. *N. ictus* LV 2006 clones are from a previous study [12]. GenBank accession numbers are given in parentheses. Maximum Likelihood/Neighbor-Joining bootstrap values greater than 50% are listed next to the respective nodes, and the bootstrap value for clade T2 is also indicated. The dashed line encloses those *Thiothrix* sequences obtained from Frasassi microbial mats.

Sequences obtained from clone libraries were compared to sequences in the public GenBank database using nucleotide BLAST [23]. 78 sequences were found to be closely related to sequences of cultivated *Thiothrix* species and to sequences previously obtained from *N. ictus* and *Thiothrix*-dominated microbial mats in Frasassi. They were used for phylogenetic analyses together with 47 closely related *Thiothrix* sequences downloaded from GenBank. All sequences were aligned using the MAFFT version 6 multiple sequence alignment tool [24] implemented with the Q-INS-I strategy for consideration of RNA secondary structure [25]. The alignment was manually refined, and a 50% consensus filter was applied in MOTHUR [26], resulting in 1369 nucleotide positions used for phylogenetic analysis. jModelTest version 0.1.1 [27] was used to determine the best-suited nucleotide model among 88 possible models following the Bayesian Information Criterion. The selected model (GTR+G) was used to build a Maximum Likelihood (ML) phylogenetic tree (1000 bootstrap replicates) using PhyML 3.0 [28]. The ML tree was rooted with an epibiont clone sequence from the hydrothermal vent galatheid crab *Shinkaia crosnieri* (GenBank accession number AB476284; [29]). In addition, Neighbor-Joining (NJ) bootstrap values for all nodes were calculated based on the same alignment using the BioNJ algorithm (Kimura 2-parameter model; 1000 bootstrap replicates) implemented in SeaView version 4 [30]. The resulting *Thiothrix* phylogenetic tree showed that most of the *Niphargus* epibiont sequences clustered into three distinct clades, which were named T1, T2, and T3 (Figure 2).

### Fluorescence *In Situ* Hybridization (FISH)

Based on sequences obtained from the 16S rDNA clone libraries, oligonucleotide FISH probes specific to *Thiothrix* clades T1–T3 (Figure 2) were designed and evaluated as described in [31]. Using PRIMROSE [32], the probes were checked against other publicly available sequences, especially those associated with Frasassi. Helper probes [33] served for increasing the chance of hybridization to poorly accessible target sites within the 16S rRNA, and competitor probes [31] were designed to prevent probe binding to other, non-target *Thiothrix* ectosymbiont sequences. All probes used in this study (see Table S1 for a list of corresponding sequences) were synthesized at Eurofins MWG Operon (Ebersberg, Germany).

FISH probes specific to T1–T3, fluorescently labeled with either fluorescein isothiocyanate (FITC) or cyanine 3 (cy3), were mixed with equimolar amounts of unlabeled competitor and helper probes to make the probe mixes NSPT1mix–NSPT3mix. To determine optimal hybridization stringencies, a FITC-labeled competitor probe with one mismatch to the respective target sequence was added to each probe mix containing a cy3-labeled clade-specific probe. Formamide concentrations were increased stepwise until the green fluorescence signal from the competitor probe disappeared and only the red signal from the clade-specific probe was detected.

33 *Niphargus* individuals and eight microbial mat samples collected between 2008 and 2011 from nine different Frasassi cave locations were examined using the T1–T3 clade-specific FISH probes. *Niphargus* and microbial mat samples for FISH were fixed in 4% paraformaldehyde for 3 h at 4°C, transferred to a 1∶1 ethanol-PBS solution, and stored at −20°C until analysis. Several legs of each *Niphargus* individual were dissected, transferred to an eppendorf vial with 100 µL of 1× PBS, and sonicated for 1 min to release the epibionts. Droplets of bacterial suspensions (epibionts or mat bacteria) were applied onto objective slides, and hybridization was carried out for 1.5 h as described in [34]. Additionally, hybridization of entire *Niphargus* legs was carried out in eppendorf tubes. Since all probe mixes had optimal hybridization stringencies of 45%, two probe mixes could be applied at a time to the same sample. Furthermore, a general bacterial EUBmix probe [35] was applied in combination with NSPT1mix, NSPT2mix, and NSPT3mix. Samples were mounted with Citifluor (Agar Scientific, Essex, UK) and examined under a Zeiss Axioplan microscope. Whole *Niphargus* legs subjected to FISH were mounted with Vectashield (Vector Laboratories, Burlingame, CA, USA), and confocal epifluorescence micrographs of attached bacteria were collected on a Zeiss 510 Meta laser scanning microscope equipped with argon and helium-neon lasers (488 and 543 nm).

### Automated Ribosomal Intergenic Spacer Analysis (ARISA) & 16S-ITS clone library construction

ARISA detects length variations in the hypervariable bacterial internal transcribed spacer (ITS) region [36]. 40 *Niphargus* individuals collected in 2008 and 2009 from eight different cave locations were examined using this molecular fingerprinting technique. ARISA-PCR was conducted as described in [37]. All DNA samples were analyzed in triplicate. Preparation for capillary electrophoresis separation and analyses of ARISA profiles were done as described in [38]. Bin frames of 2 base pairs (bp) window size and a shift window of 1.4 bp were selected by automatic binning [39]. ARISA triplicate profiles were combined so that only operational taxonomic units (OTUs) occurring in at least two of the three replicates were kept to define the final consensus profiles.

In order to identify OTUs in the ARISA profiles belonging to *Thiothrix* clades T1–T3, 16S-ITS clone libraries of *Niphargus*-associated epibiont communities were constructed. DNA extracted from three individuals of each *Niphargus* species (*N. frasassianus* from cave locations ST, RS, PC; *N. ictus* from cave locations LV, LC, PC; *N. montanarius* from cave location BG) was PCR-amplified using the tailored universal forward primer 520F (modified after [40]; complementary to *E. coli* positions 520 to 534 of the 16S rRNA) and the bacterial domain-specific reverse primer ITSReub ([41]; complementary to *E. coli* positions 23 to 37 of the 23S rRNA). The PCR mixture (50 µL) contained 1× PCR buffer (Promega, Madison, WI, USA), 1.5 mM MgCl_2_ (Promega), 0.25 mM dNTP mix (Promega), 1.5 mL bovine serum albumine (3 µg/µL), 20–25 ng of extracted DNA (quantified by a ND-1000 Nanodrop, PEQLAB), 2.5 units of GoTaq DNA polymerase (Promega), and 400 nM of each primer. PCR conditions were as follows: initial denaturation at 94°C for 3 min, followed by 30 cycles of 94°C for 45 s, 57°C for 45 s, 72°C for 90 s, and a final extension at 72°C for 5 min.

For each PCR, we used a set of three-nucleotide tags conjugated with the 5′ ends of forward and reverse primers in order to use the mark–recapture cloning method [42]. PCR products from individuals of the same *Niphargus* species were pooled before cloning, and the 5′ tags enabled identification of the *Niphargus* individual from which the respective sequence was obtained. Partial 16S-ITS sequences were assembled and manually checked for ambiguities with CodonCode Aligner version 3.7.1.1, and were submitted to Genbank (accession numbers JQ217431–JQ217456). ITS sequences belonging to *Thiothrix* clades T1–T3 were identified based on the adjoining 16S rDNA partial sequences, and their lengths were determined as distances between the target sites of the ARISA-PCR forward and reverse primers.

### Statistical analyses

Taking only the ARISA OTUs corresponding to T1–T3 *Thiothrix* into consideration, pairwise similarities among *Niphargus* samples were calculated based on the Bray-Curtis index of dissimilarity [43]. The resulting matrix was used to examine patterns in *Thiothrix* distribution among the three *Niphargus* hosts via Non-Metric Multidimensional Scaling (NMDS). NMDS places all samples in a two-dimensional coordinate system so that the ranked dissimilarities between the samples are preserved, and a stress function measures how well the original ranked distances fit into the reduced ordination space [44]. Analyses of similarities (ANOSIM) were performed to test for significant differences between predefined groups of samples (here *N. frasassianus*, *N. ictus*, and *N. montanarius*) using 1000 Monte-Carlo permutation tests. The resulting test statistic R indicates the degree of separation, ranging from 0 (no separation) to 1 (complete separation). As multiple comparisons were performed, significant ANOSIM R values were identified at the Bonferroni-corrected level (p<0.05/k, with k = n(n−1)/2, k representing the number of pair-wise comparisons between n samples). All analyses were implemented within the statistical R environment [45] using the vegan package [46] and custom R scripts [39].

### Nested-PCR assays

PCR primers specific to *Thiothrix* clades T1–T3 (Table S1) were designed based on the corresponding 16S-ITS sequences and used to screen 40 *Niphargus* individuals collected in 2008 and 2009 from eight different cave locations and all eight microbial mat samples previously investigated with FISH. A nested-PCR approach was used to increase the sensitivity of the screenings (Figure S1). In a first PCR round, bacterial 16S rDNA and ITS sequences were amplified by using the bacterial domain-specific primers 27F and ITSReub. Using the products of the first PCR as templates, a second PCR round was performed using either the *Thiothrix*-specific forward primer THIO714F or the clade-specific forward primers T2_1246F and T3_841F, as appropriate, in combination with clade-specific ITS reverse primers.

Nested-PCR was also applied to obtain partial 16S sequences of those free-living *Thiothrix* bacteria previously marked by the T2-specific FISH probe NSPT2 and to compare them with T2 sequences in 16S clone libraries of *N. ictus* and *N. montanarius*. Again using products of the first PCR round as templates, a third PCR was performed with Frasassi microbial mat samples collected in 2011 using the bacterial domain-specific forward primer 27F in combination with the clade T2-specific 16S reverse primer T2_1244R (whose sequence was congruent with that of FISH probe NSPT2).

PCR mixtures (20 µL) contained 1× ammonium buffer (Bioline), 2 mM MgCl_2_ (Bioline), 0.2 mM dNTP mix (Bioline), 2 µL of DNA extract (5–15 ng/µL; for the first PCR) or 2 µL of first PCR products (for the second and third PCR), 0.5 units of BioTaq DNA polymerase (Bioline), and 500 nM of each primer. PCR cycling conditions were identical with those used for 16S rDNA clone library construction, except for a primer annealing temperature of 56°C for the second and third PCR rounds. PCR products were checked on a 1% agarose gel, and bands of the expected size were excised and purified using the QIAquick Gel Extraction Kit (QIAGEN). Purified products were sequenced as described above. PCR sequences were compared with T1, T2, and T3 sequences previously obtained from 16S rDNA and 16S-ITS clone libraries and submitted to GenBank (accession numbers JX435482–JX435601).

16S rDNA clone libraries of *N. ictus* did not contain any sequences that clustered within *Thiothrix* clade T3 (Figure 2). However, T3 *Thiothrix* were detected on *N. ictus* individuals using FISH, ARISA, as well as PCR screenings followed by sequencing. In order to compare T3 sequences between the three *Niphargus* species, a second phylogenetic tree was constructed using the portion of the 16S rDNA sequences amplified by the T3-clade specific primers (Figure S2).

## Results and Discussion

### Diversity of *Thiothrix* ectosymbionts of Frasassi-dwelling *Niphargus* species

**Figure 3:**
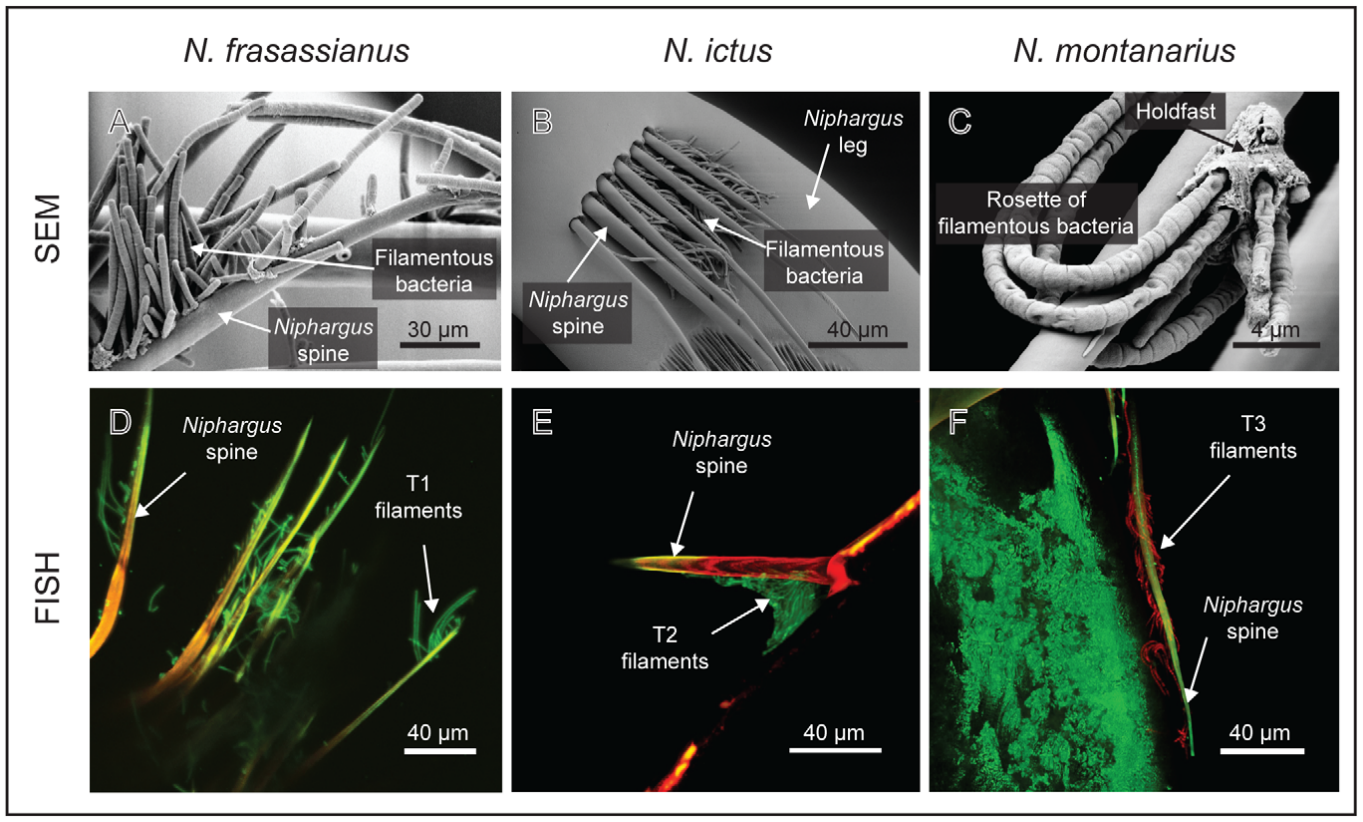
SEM and FISH images of *Thiothrix* filaments on Frasassi-dwelling *Niphargus* species. Panels A–C: Scanning electron micrographs (SEM) of bacterial filaments attached to hairs and spines on appendages of the three *Niphargus* species. The arrangement of the filaments in rosettes and their attachment via holdfasts (Panel C) are characteristic features of the bacterial genus *Thiothrix* [56]. Panels D–F: Confocal epifluorescence micrographs of *Thiothrix* filaments subjected to Fluorescence *In Situ* Hybridization (FISH) using probes specific to T1–T3 ectosymbiotic clades. Probes NSPT1 and NSPT2, designed to bind to T1 and T2, respectively (Panels D and E), were labeled with the fluorescent dye FITC (in green), T3-specific probe NSPT3 was labeled with cy3 (in red; Panel F).

The Frasassi-dwelling *Niphargus* were found to be associated with a bacterial epibiont community dominated by *Thiothrix*. SEM examination revealed that individuals of all three *Niphargus* species harbored filamentous bacteria which were attached via holdfasts to hairs and spines on the hosts' legs and antennae (Figure 3). The quantity of filaments varied between individual hosts. While both examined *N. frasassianus* specimens and the only inspected *N. montanarius* individual harbored abundant, long bacterial filaments often arranged in rosettes (Figure 3, panels A and C), three out of nine investigated *N. ictus* individuals carried only very few, solitary filaments.

Eight out of the nine 16S rDNA clone libraries constructed from *Niphargus* individuals contained *Thiothrix* sequences in different percentages (*N. frasassianus* from GB 27%, from PC 24%, from RS 70% and 54%, from ST 92%; *N. ictus* from BG 0%, from LC 60%; *N. montanarius* from BG 100% and 100%). A majority of these *Thiothrix* sequences (84%) clustered into three different phylogenetic clades, referred to as T1, T2, and T3 (Figure 2). Clade T1, supported by a 99% ML bootstrap value, contained only *Thiothrix* sequences obtained from *N. frasassianus*. Clade T2 (bootstrap value 36) was formed by *Thiothrix* sequences from *N. ictus* individuals analyzed in the present as well as in a previous study [12] and by sequences obtained from *N. montanarius*. Clade T3 (99% ML bootstrap support) was comprised of *Thiothrix* sequences from *N. montanarius* and *N. frasassianus*. T1–T3 were considered to be *Niphargus* symbiont clades, as sequences in these groups were found consistently in clone libraries from several *Niphargus* individuals collected in 2006, 2008, and 2009 from seven different cave sites.

Some *Thiothrix* sequences from *N. frasassianus* and *N. montanarius* clone libraries did not fall within the clades T1–T3, but instead clustered with *Thiothrix* sequences from Frasassi microbial mats (Figure 2). Each of these sequences was found either only once or on a single *Niphargus* individual. Moreover, using FISH we found that all filamentous bacteria attached to *Niphargus* appendages bound to one of the T1–T3 clade-specific probes NSPT1–NSPT3 (Figure 3, panels D–F). We thus concluded that the additional *Thiothrix* sequences belonged to rare epibionts or to free-living *Thiothrix* that contaminated the *Niphargus* samples during collection. Sequences belonging to other types of bacteria were also obtained in clone libraries from several *N. ictus* and *N. frasassianus* individuals (Table S2). None of these additional non-*Thiothrix* sequences was found consistently on different *Niphargus* of the same species, providing insufficient evidence for them to be regarded as symbionts.

**Figure 4:**
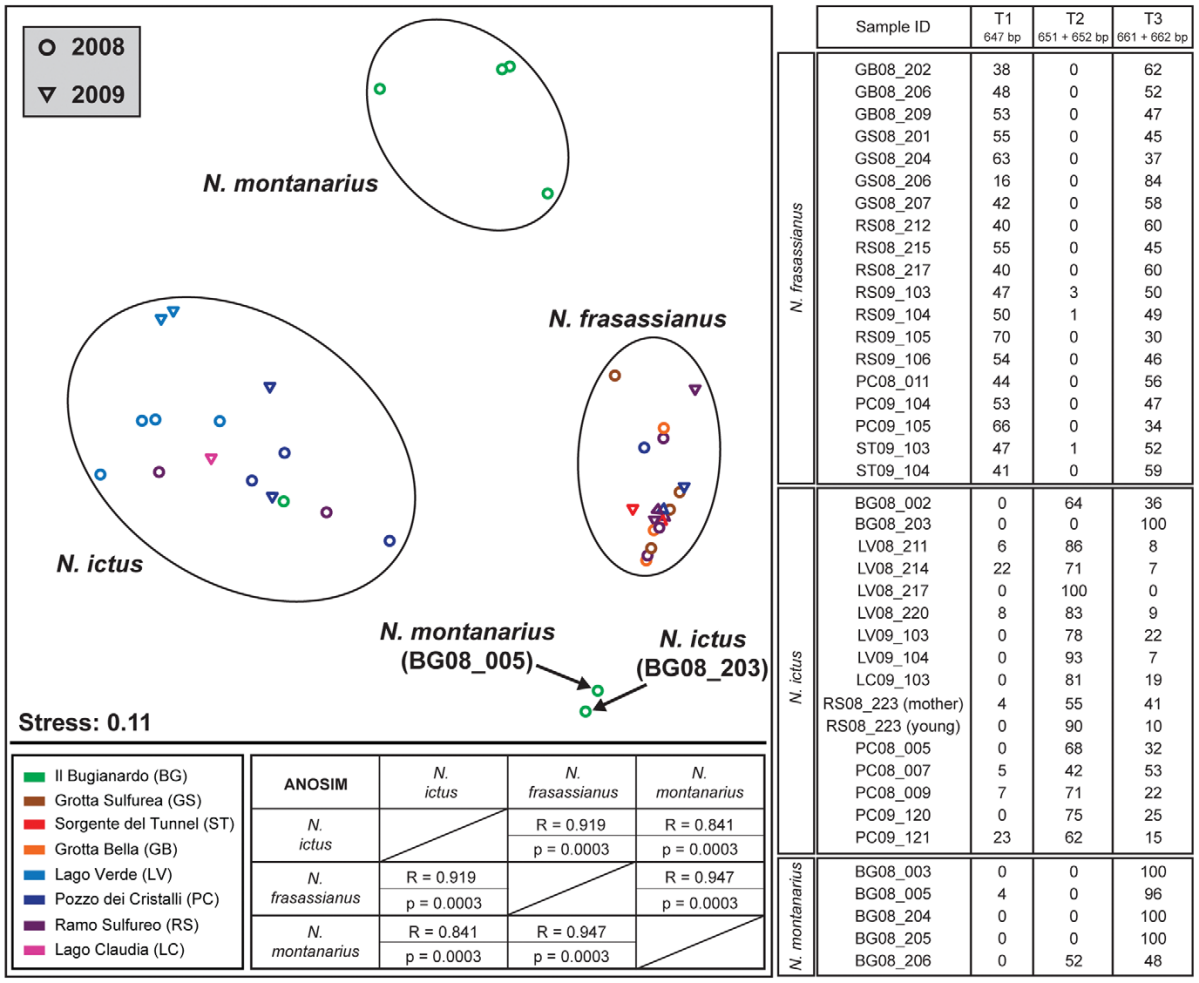
Graphical and tabular presentation of ARISA results. Left: Non-metric multidimensional scaling (NMDS) plot, in which each data point represents the *Thiothrix* ectosymbiont community structure associated with one *Niphargus* individual. Circles represent samples collected in May–June 2008 and triangles those collected in May–June 2009 from one of the eight Frasassi cave locations represented by different colors. Pairwise similarities among the *Thiothrix* ectosymbionts of *N. frasassianus*, *N. ictus*, and *N. montanarius* show that their community structures on the three host species are clearly distinct (overall ANOSIM R value: 0.894, overall Bonferroni-corrected p-value: 0.0001). Right: The table shows relative proportions (% among each other) of OTUs assigned to *Thiothrix* symbionts T1–T3 in ARISA consensus profiles of *Niphargus*-associated epibiont communities. Sample IDs indicate the sampling location and year (GB = Grotta Bella, GS = Grotta Sulfurea, RS = Ramo Sulfureo, PC = Pozzo dei Cristalli, ST = Sorgente del Tunnel, BG = Il Bugianardo, LV = Lago Verde, LC = Lago Claudia; for example, GB08_202 indicates that the sample with internal number 202 was collected from cave location GB in 2008).

16S rDNA clone libraries suggested that T1 ectosymbionts are solely harbored by *N. frasassianus*, T2 by *N. ictus* and *N. montanarius*, and T3 by *N. frasassianus and N. montanarius* (Figure 2). However, only *N. ictus* individuals collected from cave locations LV and LC were used for clone library construction. FISH analyses confirmed the results obtained from the clone libraries, but additionally revealed T3 filaments on *N. ictus* individuals from cave locations BG and PC (Table 1). Moreover, ARISA as well as nested-PCR detected the presence of T3 *Thiothrix* on *N. ictus* from all sampled cave locations, including LV and LC (Figure 4; Table 1). This discrepancy between the results of the different methods might be explained by very low amounts of T3 *Thiothrix* cells on *N. ictus* individuals from LV and LC, which might have been sufficient to be detected by highly sensitive techniques like ARISA and nested-PCR, but insufficient to become represented in 16S rDNA clone libraries or be revealed by FISH. The overall evidence suggests that T3 *Thiothrix* are ectosymbionts of all three Frasassi-dwelling *Niphargus* species.

**Table 1:** Results of FISH experiments and nested-PCR assays on *Niphargus* individuals.

*Niphargus* individuals			FISH results				PCR results			
Species	Cave	Year	Individuals	# of individuals harboring			Individuals	# of individuals		
			analyzed	filaments that bound to			analyzed	containing		
			(n)	NSPT1	NSPT2	NSPT3	(n)	T1	T2	T3
*Niphargus*	GS	2008	0	n.a.	n.a.	n.a.	4	4	3	4
*frasassianus*		2009	1	1	0	1	0	n.a.	n.a.	n.a.
	ST	2009	2	2	0	2	2	2	2	2
		2010	6	6	0	6	0	n.a.	n.a.	n.a.
	GB	2008	0	n.a.	n.a.	n.a.	3	3	3	3
		2009	1	1	0	1	0	n.a.	n.a.	n.a.
	PC	2008	0	n.a.	n.a.	n.a.	1	1	1	1
		2009	3	3	0	3	2	2	2	2
	RS	2008	0	n.a.	n.a.	n.a.	3	3	3	3
		2009	0	n.a.	n.a.	n.a.	4	4	4	4
*Niphargus*	BG	2008	0	n.a.	n.a.	n.a.	2	0	2	2
*ictus*		2011	2	0	2	2	0	n.a.	n.a.	n.a.
	LV	2008	0	n.a.	n.a.	n.a.	4	0	4	4
		2009	1	0	1	0	2	0	2	1
		2010	1	0	1	0	0	n.a.	n.a.	n.a.
		2011	6	0	6	0	0	n.a.	n.a.	n.a.
	PC	2008	0	n.a.	n.a.	n.a.	3	2	3	3
		2009	2	0	2	1	2	2	2	2
	RS	2008	0	n.a.	n.a.	n.a.	2	1	2	2
	LC	2009	0	n.a.	n.a.	n.a.	1	0	1	1
*Niphargus*	BG	2008	0	n.a.	n.a.	n.a.	5	2	5	5
*montanarius*		2010	6	0	6	6	0	n.a.	n.a.	n.a.
		2011	1	0	1	1	0	n.a.	n.a.	n.a.

NSPT1–NSPT3 = *Niphargus* Symbiont Probes specific to *Thiothrix* clades T1–T3. GS = Grotta Sulfurea, ST = Sorgente del Tunnel, GB = Grotta Bella, PC = Pozzo dei Cristalli, RS = Ramo Sulfureo, BG = Il Bugianardo, LV = Lago Verde, LC = Lago Claudia. n.a. = not analyzed.

### Origins of the *Niphargus*-*Thiothrix* ectosymbioses

Closest relatives of ectosymbionts T1 and T2 are *Thiothrix* from Frasassi cave microbial mats (Figure 2). Thus, it is likely that T1 and T2 symbionts have evolved within the cave system from previously free-living bacteria. Due to poor bootstrap support for nodes connecting clades T1 and T2, it is not possible to say whether the ectosymbionts evolved from a common symbiotic ancestor or from distinct free-living *Thiothrix*. The three Frasassi-dwelling *Niphargus* species probably invaded the cave system independently, but in a yet unknown order [17]. Therefore, it is currently not possible to speculate about the sequence in which T1 and T2 symbionts were acquired by their different hosts.

The T3 clade is distantly related to clades T1 and T2 and to all other Frasassi cave microbial mat sequences. Consequently, T3 ectosymbionts seem to have originated from outside the Frasassi cave system. The three *Niphargus* species investigated in this study are distantly related to each other [17], and each of them harbors T3 symbionts. This suggests that T3 symbionts are either widespread in the genus *Niphargus*, or were introduced into Frasassi by one *Niphargus* host and subsequently dispersed over the remaining species inside the caves. Investigations of non-Frasassi *Niphargus* species for *Thiothrix* ectosymbionts are currently underway to evaluate these alternatives.

Our analyses suggest that the *Niphargus*-*Thiothrix* symbiosis has evolved independently at least two times. Symbioses with T1 and T2 may have been initiated within the last one million years, during which sulfidic conditions within the Frasassi caves have been established [12], whereas the association with T3 is likely more ancient.

### *Thiothrix* ectosymbiont transmission mode

**Figure 5:**
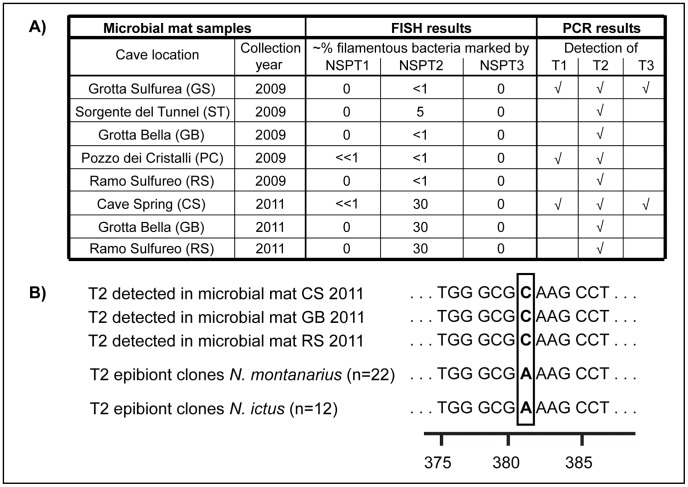
Results of FISH and PCR analyses of Frasassi microbial mats. (A) NSPT1–NSPT3 = *Niphargus* Symbiont Probes specific to T1–T3. √ = PCR product of expected size verified by sequencing to belong to clades T1, T2, or T3. (B) Consistent one-base difference between T2 16S rDNA sequences derived from microbial mats and *Niphargus* samples. n = number of clones. Numbers below the sequences refer to 16S rRNA nucleotide positions according to *Escherichia coli* numbering [57]. Microbial mat T2 sequences were obtained by sequencing PCR products using T2-specific primers, whereas T2 epibiont sequences were obtained from 16S rDNA clone libraries of *Niphargus*.

FISH with oligonucleotide probes specific to T1–T3 was used to examine whether significant free-living populations of the ectosymbionts were present in Frasassi cave microbial mats. These analyses revealed that T1 filaments were nearly absent from the mats (Table 1), except for two short filaments (both <10 µm) observed in two samples. Free-living *Thiothrix* filaments tend to be several 100 micrometers in lengths, while ectosymbiont filaments on *Niphargus* appear to be “groomed” to lengths between 30 and 100 µm (Figure 3). It is thus likely that the short T1 filaments detected within the mat samples were shed ectosymbionts rather than steady members of the microbial mat community. T2-specific probe NSPT2 bound to few filaments in mats collected before or in the year 2009 ([12]; Figure 5), but to considerably more filaments in mats collected in 2011. T3 filaments were not detected in any of the eight Frasassi microbial mat samples analyzed by FISH (Figure 5).

Bacterial symbionts can either be transmitted horizontally, where they get repeatedly recruited from the host's environment, or vertically, where they are passed down from one host generation to the next [20]. Considering the dearth of T1 and T3 free-living counterparts in Frasassi microbial mats, horizontal transmission through the perpetual reacquisition of these symbionts from the mats appears doubtful. In the *Thiothrix* phylogenetic tree (Figure 2), a relatively long branch separates clade T1 from Frasassi microbial mat sequences, and the T3 clade is very distinct from all sequences obtained from Frasassi. Both clades are also strongly supported by ML bootstrap values of 99%. This is consistent with the increased genetic isolation and homogeneity that accompanies vertical symbiont transmission [20], [47]–[48].

FISH revealed a significant free-living population of T2 in Frasassi cave microbial mats collected in 2011 (Table 1). However, 16S rDNA sequences amplified from the 2011 mat samples using T2-specific PCR primers had a consistent one-base difference to all sequences derived from *N. ictus* and *N. montanarius* (Figure 5). From this it follows that T2 ectosymbionts of *N. ictus* and *N. montanarius* are very closely related to but nevertheless distinct from the filamentous mat bacteria marked by probe NSPT2. This indicates a lack of interchange between the free-living and ectosymbiotic T2 communities, suggesting that horizontal transmission is not prevalent.

16S rDNA sequences of T2 symbionts derived from *N. ictus* and *N. montanarius* are indistinguishable. This suggests that these symbionts freely alternate between their respective *Niphargus* hosts. In the case of T3, three distinct types of 16S rDNA sequences were obtained: one derived from only one *N. montanarius* individual, a second from several individuals of all three *Niphargus* species, and a third from several individuals of *N. ictus* and one *N. montanarius* individual (Figure S2). It is possible that the three subtypes of T3 were introduced into Frasassi by the three *Niphargus* species and two of them were subsequently exchanged among different hosts. Taken together, the evidence indicates that T2 and T3 symbionts are transmitted among different hosts using a combination of intra- and interspecific inoculations, whereas T1 symbionts are transmitted exclusively among *N. frasassianus*.

Vertical transmission has been commonly reported for endosymbioses where symbionts are passed down through the host female germ line [20]. A “pseudo-vertical” transmission mode was previously suggested for *N. ictus*, whereby symbionts are transmitted externally onto eggs or juveniles carried in the female's brood-pouch [12]. The *Niphargus* sample set used for ARISA (Figure 4) included a *Niphargus* baby (“RS08_223 (young)”) that had been removed from the brood pouch of the mother animal (“RS08_223 (mother)”) just before DNA extraction. ARISA results revealed that the juvenile individual, like its mother, harbored T2 and T3 ectosymbionts on its appendages.

*Thiothrix* bacteria can relocate themselves through gonidia, which are motile dispersal cells produced from the apex of the filaments [49]–[50]. Nested-PCR assays detected T1, T2 and T3 in many Frasassi microbial mats, including samples where FISH had indicated an absence of full-grown filaments (Figure 5). The highly sensitive PCR analyses presumably detected *Thiothrix* gonidia that are present in Frasassi cave waters. These gonidia may be the means by which T1–T3 ectosymbionts are exchanged among *Niphargus* hosts living in various cave locations throughout Frasassi.

### Host-specific *Thiothrix* distribution patterns

ARISA served to identify compositional differences in the *Thiothrix* communities associated with the three *Niphargus* species. Determining the ITS lengths of the *Thiothrix* symbionts from 16S-ITS clone libraries allowed us to assign individual OTUs in the ARISA consensus profiles to T1, T2, and T3. Symbiont ITS lengths were as follows: 647 bp (T1), 651, 652 bp (T2), and 661, 662 bp (T3). Taking only the ARISA OTUs corresponding to these five ITS sequence lengths into account for NMDS analysis, calculated pairwise similarities confirmed that the relative distribution patterns of *Thiothrix* symbionts on the three *Niphargus* species were host species-specific (Figure 4). Except for two outliers (*N. ictus* BG08_203 and *N. montanarius* BG08_005, which were both dominated by the 662bp T3 OTU), all analyzed *Niphargus* samples clustered into three different groups according to their respective host species. This was the case even for *Niphargus* individuals of two different species that co-occurred and were collected simultaneously at the same cave locations (BG, PC, RS). *N. ictus* and *N. frasassianus* inhabit several different cave sites where the dominant microbial mat type varies based on the cave water geochemistry and flow regime (Figure 1). However, neither sampling location nor collection year had a major influence on the relative *Thiothrix* ectosymbiont distribution patterns on these hosts. Despite the two outliers, ANOSIM statistics (R and p values) confirmed clear separation with high significance between the three *Niphargus* species. It is however important to note that the sample size of *N. montanarius* (n = 5) was much smaller than that of *N. ictus* (n = 16) and *N. frasassianus* (n = 19). Unequal sample sizes lower the meaningfulness of ANOSIM, but were unavoidable in our study due to the low availability of *N. montanarius* individuals.

T1 and T3 OTUs were abundant on *N. frasassianus*, whereas *N. ictus* and *N. montanarius* were mainly associated with T2 and T3. This is in close agreement with our FISH results (Table 1). The clear separation between the three *Niphargus* species in the NMDS plot generated from ARISA (Figure 4) was obtained using a Bray-Curtis index of dissimilarity for the calculations, which considers both presence/absence and relative abundance of *Thiothrix* OTUs. Using a Jaccard index instead, which takes only OTU presence/absence into account, resulted in non-significant ANOSIM values and poor separation between the three groups in the NMDS plot. Close examination showed that T2 ARISA OTUs were also occasionally detected on *N. frasassianus*, and T1 OTUs on *N. ictus* and *N. montanarius*. Thus, the ARISA results indicate that T1–T3 *Thiothrix* ectosymbionts can colonize all three *Niphargus* hosts, but their abundances are strongly influenced by the identity of the host species they are associated with. This is consistent with the comparison between FISH and PCR results, where FISH shows a host-species specific distribution pattern, whereas nested-PCR assays detected T1–T3 sequences on all three *Niphargus* species (Table 1). While the highly sensitive nested-PCR approach might trace gonidia attached to *Niphargus* exoskeletons, FISH would only reveal full-grown *Thiothrix* filaments. Taken together, our analyses suggest that T1–T3 ectosymbiont gonidia can attach to exoskeletons of all three *Niphargus* species. However, only T1 and T3 filaments develop successfully on *N. frasassianus*, whereas T2 and T3 flourish on *N. ictus* and *N. montanarius*, with T3 dominating *N. montanarius* ectosymbiont communities.

Chitin, the major component of *Niphargus* exoskeletons, is a common binding motif for many invertebrate-microbe associations [19]. *Thiothrix* belonging to the clades T1, T2, and T3 all appear to have the ability and the preference to colonize the chitinous surfaces of *Niphargus* amphipods, but their host-species specific distribution pattern is likely caused by factors controlled by each *Niphargus* species.

Selection of particular ectosymbiont clades may be mediated by lectins secreted on the *Niphargus* cuticle. Such a mechanism has previously been shown to enable differential ectosymbiont acquisition by nematodes dwelling in sulfidic marine sediments [10], [51]. Another possibility is that the three *Thiothrix* ectosymbionts have different tolerances and requirements for sulfide and oxygen. Thus, their respective prevalences on different *Niphargus* species may result from their adaptation to the distinct locomotion behaviors and microhabitat preferences of their hosts. *N. ictus* is a swimming species that prefers stagnant, stratified water bodies. It travels between oxygenated top layers and sulfidic bottom zones and thereby exposes its ectosymbionts to alternating redox conditions [12], [17]. *N. frasassianus* is a poor swimmer and favors shallow, turbulent streams, where it crawls among sulfide-rich sediments and microbial mats [17]. Thus, its ectosymbionts are continuously and simultaneously provided with sulfide and oxygen. *N. montanarius* is found exclusively in cave location BG [17], which is a non-sulfidic pool (Figure 1). We are currently examining the metabolic capabilities of the three *Thiothrix* ectosymbionts to infer the benefits that each of them may derive from its particular ‘hitch-hiking’ lifestyle.

### Conclusion

While symbioses between invertebrates and sulfur-oxidizing bacteria have been extensively studied in the marine environment [2], [52], the first example from a freshwater ecosystem involving *Niphargus* amphipods was discovered only recently [12]. In this study, we found that three *Niphargus* species living in the sulfide-rich cave system of Frasassi are ectosymbiotic with filamentous *Thiothrix* ectosymbionts of three different clades. The genus *Niphargus* contains over 300 species distributed throughout Europe [53]–[54], some of which are found in other sulfidic locations, such as Movile cave in Romania [55]. *Niphargus*-*Thiothrix* ectosymbioses are thus potentially widespread in the subterranean realm and warrant further investigation.

## Supporting Information

Figure S1 16S rDNA and ITS binding sites of *Thiothrix* clade-specific PCR primers.
(PDF)

Figure S2 16S Maximum Likelihood phylogenetic tree of *Thiothrix* clade T3, including all sequences obtained from 16S clone libraries and nested-PCR assays. Colors mark the different sources from which the sequences were obtained (red = *N. frasassianus*, blue = *N. ictus*, green = *N. montanarius*, brown = Frasassi microbial mats). Clone and sequence names indicate the sampling location and year (BG = Il Bugianardo, GS = Grotta Sulfurea, CS = Cave Spring, ST = Sorgente del Tunnel, GB = Grotta Bella, LV = Lago Verde, PC = Pozzo dei Cristalli, RS = Ramo Sulfureo, LC = Lago Claudia). Different numbers after cave name abbreviations indicate different *Niphargus* individuals collected from the same cave location (cf. Figure 2). GenBank accession numbers are given in parentheses. Maximum Likelihood/Neighbor-Joining bootstrap values greater than 50% are listed next to the respective nodes.
(PDF)

Table S1 List of all FISH probes and PCR primers used in this study.
(XLS)

Table S2 List of non-*Thiothrix* sequences obtained from 16S rDNA clone libraries of *Niphargus*-associated epibionts.
(PDF)
