# Supplementary material for: Repeatedly Evolved Host-Specific Ectosymbioses between Sulfur-Oxidizing Bacteria and Amphipods Living in a Cave Ecosystem
Source: PLoS One. 2012 Nov 29;7(11):e50254. doi: 10.1371/journal.pone.0050254 (PMC3510229; doi:10.1371/journal.pone.0050254)
Supplement: Figure S1 — 16S rDNA and ITS binding sites of Thiothrix clade-specific PCR primers. (PDF) [file pone.0050254.s001.pdf]

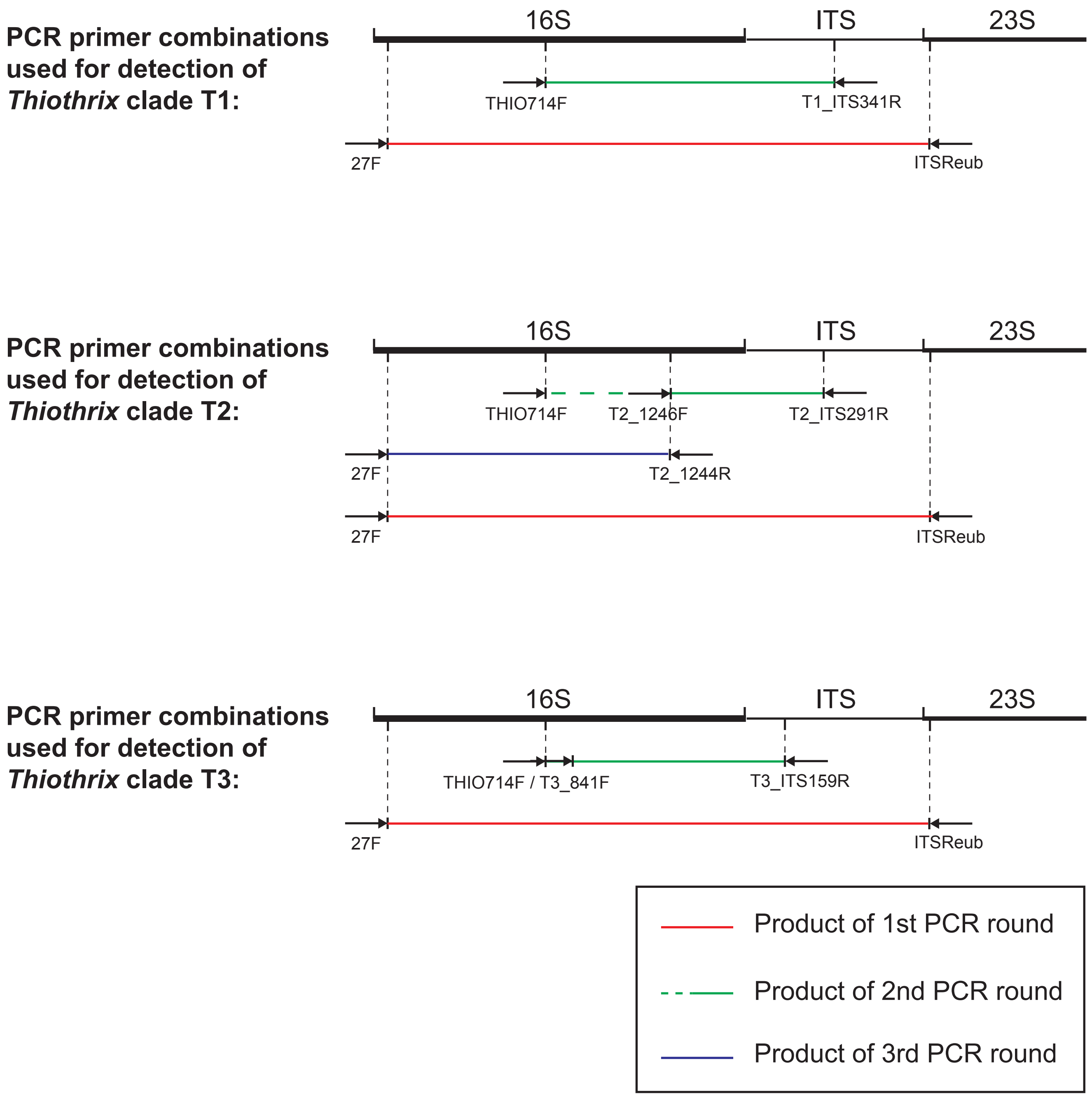
PCR primer combinations used for detection of *Thiothrix* clade T1:
16S
ITS
23S
THIO714F
T1_ITS341R
27F
ITSReub
PCR primer combinations used for detection of *Thiothrix* clade T2:
16S
ITS
23S
THIO714F
T2_1246F
T2_ITS291R
27F
T2_1244R
27F
ITSReub
PCR primer combinations used for detection of *Thiothrix* clade T3:
16S
ITS
23S
THIO714F / T3_841F
T3_ITS159R
27F
ITSReub
Product of 1st PCR round
Product of 2nd PCR round
Product of 3rd PCR round
