# Supplementary material for: Repeatedly Evolved Host-Specific Ectosymbioses between Sulfur-Oxidizing Bacteria and Amphipods Living in a Cave Ecosystem
Source: PLoS One. 2012 Nov 29;7(11):e50254. doi: 10.1371/journal.pone.0050254 (PMC3510229; doi:10.1371/journal.pone.0050254)
Supplement: Figure S2 — 16S Maximum Likelihood phylogenetic tree of Thiothrix clade T3, including all sequences obtained from 16S clone libraries and nested-PCR assays. Colors mark the different sources from which the sequences were obtained (red = N. frasassianus, blue = N. ictus, green = N. montanarius, brown = Frasassi microbial mats). Clone and sequence names indicate the sampling location and year (BG = Il Bugianardo, GS = Grotta Sulfurea, CS = Cave Spring, ST = Sorgente del Tunnel, GB = Grotta Bella, LV = Lago Verde, PC = Pozzo dei Cristalli, RS = Ramo Sulfureo, LC = Lago Claudia). Different numbers after cave name abbreviations indicate different Niphargus individuals collected from the same cave location (cf. Figure 2). GenBank accession numbers are given in parentheses. Maximum Likelihood/Neighbor-Joining bootstrap values greater than 50% are listed next to the respective nodes. (PDF) [file pone.0050254.s002.pdf]

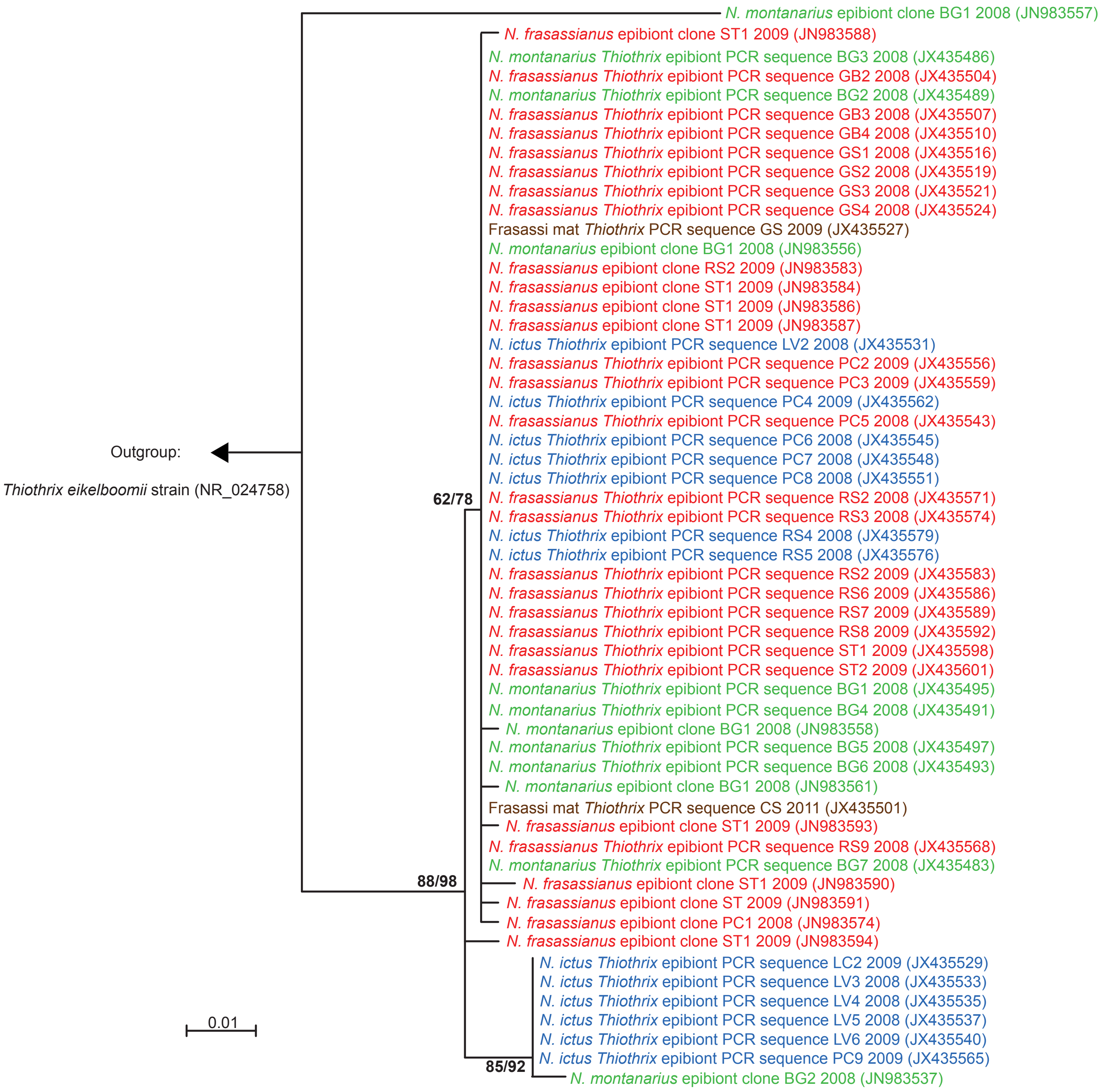
N. montanarius epibiont clone BG1 2008 (JN983557)
N. frasassianus epibiont clone ST1 2009 (JN983588)
N. montanarius Thiothrix epibiont PCR sequence BG3 2008 (JX435486)
N. frasassianus Thiothrix epibiont PCR sequence GB2 2008 (JX435504)
N. montanarius Thiothrix epibiont PCR sequence BG2 2008 (JX435489)
N. frasassianus Thiothrix epibiont PCR sequence GB3 2008 (JX435507)
N. frasassianus Thiothrix epibiont PCR sequence GB4 2008 (JX435510)
N. frasassianus Thiothrix epibiont PCR sequence GS1 2008 (JX435516)
N. frasassianus Thiothrix epibiont PCR sequence GS2 2008 (JX435519)
N. frasassianus Thiothrix epibiont PCR sequence GS3 2008 (JX435521)
N. frasassianus Thiothrix epibiont PCR sequence GS4 2008 (JX435524)
Frasassi mat Thiothrix PCR sequence GS 2009 (JX435527)
N. montanarius epibiont clone BG1 2008 (JN983556)
N. frasassianus epibiont clone RS2 2009 (JN983583)
N. frasassianus epibiont clone ST1 2009 (JN983584)
N. frasassianus epibiont clone ST1 2009 (JN983586)
N. frasassianus epibiont clone ST1 2009 (JN983587)
N. ictus Thiothrix epibiont PCR sequence LV2 2008 (JX435531)
N. frasassianus Thiothrix epibiont PCR sequence PC2 2009 (JX435556)
N. frasassianus Thiothrix epibiont PCR sequence PC3 2009 (JX435559)
N. ictus Thiothrix epibiont PCR sequence PC4 2009 (JX435562)
N. frasassianus Thiothrix epibiont PCR sequence PC5 2008 (JX435543)
N. ictus Thiothrix epibiont PCR sequence PC6 2008 (JX435545)
N. ictus Thiothrix epibiont PCR sequence PC7 2008 (JX435548)
N. ictus Thiothrix epibiont PCR sequence PC8 2008 (JX435551)
Outgroup:
Thiothrix eikelboomii strain (NR_024758)
62/78
N. frasassianus Thiothrix epibiont PCR sequence RS2 2008 (JX435571)
N. frasassianus Thiothrix epibiont PCR sequence RS3 2008 (JX435574)
N. ictus Thiothrix epibiont PCR sequence RS4 2008 (JX435579)
N. ictus Thiothrix epibiont PCR sequence RS5 2008 (JX435576)
N. frasassianus Thiothrix epibiont PCR sequence RS2 2009 (JX435583)
N. frasassianus Thiothrix epibiont PCR sequence RS6 2009 (JX435586)
N. frasassianus Thiothrix epibiont PCR sequence RS7 2009 (JX435589)
N. frasassianus Thiothrix epibiont PCR sequence RS8 2009 (JX435592)
N. frasassianus Thiothrix epibiont PCR sequence ST1 2009 (JX435598)
N. frasassianus Thiothrix epibiont PCR sequence ST2 2009 (JX435601)
N. montanarius Thiothrix epibiont PCR sequence BG1 2008 (JX435495)
N. montanarius Thiothrix epibiont PCR sequence BG4 2008 (JX435491)
N. montanarius epibiont clone BG1 2008 (JN983558)
N. montanarius Thiothrix epibiont PCR sequence BG5 2008 (JX435497)
N. montanarius Thiothrix epibiont PCR sequence BG6 2008 (JX435493)
N. montanarius epibiont clone BG1 2008 (JN983561)
Frasassi mat Thiothrix PCR sequence CS 2011 (JX435501)
N. frasassianus epibiont clone ST1 2009 (JN983593)
N. frasassianus Thiothrix epibiont PCR sequence RS9 2008 (JX435568)
N. montanarius Thiothrix epibiont PCR sequence BG7 2008 (JX435483)
88/98
N. frasassianus epibiont clone ST1 2009 (JN983590)
N. frasassianus epibiont clone ST 2009 (JN983591)
N. frasassianus epibiont clone PC1 2008 (JN983574)
N. frasassianus epibiont clone ST1 2009 (JN983594)
N. ictus Thiothrix epibiont PCR sequence LC2 2009 (JX435529)
N. ictus Thiothrix epibiont PCR sequence LV3 2008 (JX435533)
N. ictus Thiothrix epibiont PCR sequence LV4 2008 (JX435535)
0.01
N. ictus Thiothrix epibiont PCR sequence LV5 2008 (JX435537)
N. ictus Thiothrix epibiont PCR sequence LV6 2009 (JX435540)
N. ictus Thiothrix epibiont PCR sequence PC9 2009 (JX435565)
85/92
N. montanarius epibiont clone BG2 2008 (JN983537)

Clade T3 phylogenetic tree construction

16S portions (~600 bp fragments) of 41 T3 sequences obtained from PCR screenings of *Niphargus* individuals and Frasassi microbial mat samples were aligned with all T3 sequences contained in *Niphargus* 16S clone libraries using the MAFFT version 6 multiple sequence alignment tool [24] implemented with the Q-INS-I strategy for consideration of RNA secondary structure [25]. The alignment was manually refined, and a 50% consensus filter was applied in MOTHUR [26], resulting in 579 nucleotide positions used for phylogenetic analysis. jModelTest version 0.1.1 [27] was used to determine the best-suited nucleotide model among 88 possible models following the Bayesian Information Criterion. The selected model (TIM3+I) was used to build a Maximum Likelihood (ML) phylogenetic tree (1000 bootstrap replicates) using PhyML 3.0 [28]. The ML tree was rooted using a *Thiothrix eikelboomii* strain sequence downloaded from the GenBank database (accession number NR_024758). In addition, Neighbor-Joining (NJ) bootstrap values for all nodes were calculated based on the same alignment using the BioNJ algorithm (Kimura 2-parameter model; 1000 bootstrap replicates) implemented in SeaView version 4 [30].
