# Supplementary material for: Repeatedly Evolved Host-Specific Ectosymbioses between Sulfur-Oxidizing Bacteria and Amphipods Living in a Cave Ecosystem
Source: PLoS One. 2012 Nov 29;7(11):e50254. doi: 10.1371/journal.pone.0050254 (PMC3510229; doi:10.1371/journal.pone.0050254)
Supplement: Table S2 — List of non-Thiothrix sequences obtained from 16S rDNA clone libraries of Niphargus-associated epibionts. (PDF) [file pone.0050254.s004.pdf]

Bauermeister Supporting Information Table S2 (Part I)

Host species	Sampling location, year	Clone # (GenBank accession No.)	Top blastn hit / source (GenBank accession No.)	Query coverage*	Maximum identity
N. ictus	Il Bugianardo (BG), 2008	10 (JN983615)	Uncultured bacterium clone reservoir-27 / Dianchi Lake bacterioplankton community (JF697408.1)	100%	93%
		11 (JN983616)	*Propionibacterium acnes* (AB573714.1)	100%	99%
		13 (JN983617)	*Candidatus Hepatoplasma crinochetorum* clone 59 / terrestrial isopod gut symbiont (AY500250.1)	100%	87%
		14 (JN983618)	Uncultured *Staphylococcus* sp. clone EHFS1_S09d / European clean room environments (EU071495.1)	99%	99%
		16 (JN983619)	Uncultured bacterium clone Dianchi-29 / Dianchi Lakeshore polluted water microbial community (HQ324875.1)	100%	96%
		17 (JN983620)	**Uncultured Bacteroidetes bacterium clone UMAB-cl-134 / Antarctic Peninsula microbial community (FR749759.1)**	**100%**	**95%**
		19 (JN983621)	Uncultured Pseudomonadales bacterium clone LCM020 / bacterial colonalization on adult black fly (JF733452.1)	100%	94%
		20 (JN983622)	Uncultured bacterium clone reservoir-27 / Dianchi Lake bacterioplankton community (JF697408.1)	100%	94%
		21 (JN983623)	*Fangia hongkongensis* strain UST040201-002 / coastal seawater of Hongkong (NR_041041.1)	99%	82%
		23 (JN983624)	Uncultured bacterium clone lka65 / Frasassi microbial mat community (EF467563.1)	100%	99%
		25 (JN983625)	Uncultured bacterium clone DLN-150 / fecal microbiota in gayal (FJ848446.1)	100%	97%
		26 (JN983626)	Uncultured bacterium clone reservoir-27 / Dianchi Lake bacterioplankton community (JF697408.1)	100%	94%
		28 (JN983627)	*Candidatus Hepatoplasma crinochetorum* clone 59 / terrestrial isopod gut symbiont (AY500250.1)	99%	87%
		29 (JN983628)	Uncultured bacterium clone zEL43 / Frasassi microbial mat community (DQ415836.1)	100%	95%
		33 (JN983629)	Uncultured bacterium clone EDW07B005_144 / Edwards Aquifer microbial community (HM066626.1)	100%	96%
		34 (JN983630)	Uncultured Pseudomonadales bacterium clone LCM020 / bacterial colonalization on adult black fly (JF733452.1)	100%	95%
N. frasassianus	Grotta Bella (GB), 2008	16 (JN983631)	Uncultured epsilon proteobacterium clone 4-138 / Alum Rock Park springs microbial community (GQ354915.1)	100%	99%
		20 (JN983632)	Uncultured bacterium clone VC105120 / Frasassi microbial mat community (JF747849.1)	100%	99%
		21 (JN983633)	Uncultured bacterium clone FC1_16S_47 / sulfidic karst systems microbial community (EU662454.1)	100%	99%
		27 (JN983634)	**Uncultured Bacteroidetes bacterium clone UMAB-cl-134 / Antarctic Peninsula bacterial community (FR749759.1)**	**100%**	**95%**
		11 (JN983635)	**Uncultured bacterium clone EV818SWSAP25 / Kalahari Shield subsurface water bacterial community (DQ337068.1)**	**100%**	**94%**
		3 (JN983636)	Uncultured bacterium clone VC1058 / Frasassi microbial mat community (JF747820.1)	100%	99%
		4 (JN983637)	Uncultured bacterium clone C3E / Chinese mitten crab (*Eriocheir sinensis*) intestinal bacteria (DQ856548.1)	99%	93%
		6 (JN983638)	**Uncultured bacterium clone EV818SWSAP25 / Kalahari Shield subsurface water bacterial community (DQ337068.1)**	**100%**	**94%**
		8 (JN983639)	**Uncultured Bacteroidetes bacterium clone UMAB-cl-134 / Antarctic Peninsula bacterial community (FR749759.1)**	**100%**	**95%**

* Reference value for blastn hit ranking
Purple = Top nucleotide BLAST (blastn) hit matched by more than one sequence from the same clone library
Red = Top nucleotide BLAST (blastn) hit matched by several sequences from different clone libraries

Bauermeister Supporting Information Table S2 (Part II)

Host species	Sampling location, year	Clone # (GenBank accession No.)	Top blastn hit / source (GenBank accession No.)	Query coverage*	Maximum identity
N. ictus	Lago Claudia (LC), 2009	2 (JN983640)	Uncultured Bacteroidetes bacterium clone IC4044 / Arctic glacier ice bacterial community (HQ622740.1)	100%	96%
		5 (JN983641)	Unidentified proteobacterium clone BD1-5 / deep-sea sediment bacterial community (AB015518.1)	100%	90%
		6 (JN983642)	Uncultured bacterium clone 8S7 / marine biofilm microbial community (JF272066.1)	100%	88%
		8 (JN983643)	Uncultured bacterium clone FL0428B_PF27 / sulfate-reducing cave biofilm microbial community (FJ716413.1)	100%	95%
N. frasassianus	Pozzo dei Cristalli (PC), 2008	1 (JN983644)	Uncultured bacterium clone 2C228550 / aquatic bacterial community (EU800447.1)	100%	98%
		3 (JN983645)	Uncultured bacterium clone FrsFi199 / green phototrophic biofilm microbial community, Frasassi (JF747962.1)	100%	99%
		4 (JN983646)	Uncultured bacterium clone VC10574 / Frasassi microbial mat community (JF747856.1)	100%	99%
		5 (JN983647)	Uncultured bacterium clone lka79 / Frasassi microbial mat community (EF467575.1)	100%	99%
		6 (JN983648)	Uncultured bacterium clone 2C228550 / aquatic bacterial community (EU800447.1)	100%	98%
		7 (JN983649)	Uncultured bacterium clone 655952 / contaminated sediment microbial community (DQ404824.1)	100%	85%
		8 (JN983650)	Alpha proteobacterium A0904 16S / - (AF236001.1)	100%	93%
		49 (JN983651)	Uncultured bacterium clone 3BR-12AA / circumneutral freshwater microbial community (EU937911.1)	100%	96%
		51 (JN983652)	Uncultured bacterium clone SILK48 / Frasassi microbial mat community (EF467488.1)	100%	99%
		52 (JN983653)	Uncultured bacterium clone TP-SL-B-271 / Shule river permaforst bacterial community (HQ864209.1)	100%	96%
		53 (JN983654)	Uncultured bacterium clone BCWCWP1A42 / western Ghats (India) microbial community (FJ598380.1)	100%	96%
		56 (JN983655)	Uncultured bacterium clone WM4 / Frasassi mat microbial community (DQ133921.1)	100%	99%
		58 (JN983656)	**Uncultured bacterium clone A156 / wastewater treatment plant microbial community (FJ660593.1)**	**100%**	**97%**
	Ramo Sulfureo (RS), 2008	19 (JN983663)	**Uncultured bacterium clone EV818SWSAP25 / Kalahari Shield subsurface water bacterial community (DQ337068.1)**	**100%**	**94%**
		25 (JN983664)	Uncultured rumen bacterium clone L102RT-2-D06 / bovine ruminal epithelial bacterial community (HQ400111.1)	100%	82%
		33 (JN983665)	Uncultured bacterium clone CV104 / Frasassi acidic cave wall biofilm microbial community (EF530681.1)	100%	99%
	Ramo Sulfureo (RS), 2009	11 (JN983657)	Uncultured bacterium clone FGL7S_B30 / Fayetteville Green Lake microbial community (FJ437988.1)	100%	98%
		14 (JN983658)	**Uncultured bacterium clone A156 / wastewater treatment plant microbial community (FJ660593.1)**	**100%**	**98%**
		16 (JN983659)	**Uncultured bacterium clone A156 / wastewater treatment plant microbial community (FJ660593.1)**	**100%**	**97%**
		17 (JN983660)	Uncultured bacterium clone Dianchi-34 / Dianchi lakeshore polluted water microbial community (HQ324880.1)	100%	95%
		26 (JN983661)	Uncultured epsilon proteobacterium clone / sulfur spring microbial community (AB425206.1)	100%	99%
		28 (JN983662)	**Uncultured bacterium clone A156 / wastewater treatment plant microbial community (FJ660593.1)**	**100%**	**97%**
	Sorgente del Tunnel (ST), 2009	30 (JN983666)	**Uncultured Bacteroidetes bacterium clone UMAB-cl-134 / Antarctic Peninsula bacterial community (FR749759.1)**	**100%**	**95%**

* Reference value for blastn hit ranking
Purple = Top nucleotide BLAST (blastn) hit matched by more than one sequence from the same clone library
Red = Top nucleotide BLAST (blastn) hit matched by several sequences from different clone libraries
